# Evaluation of fecal sampling time points to estimate apparent nutrient digestibility in lactating Holstein dairy cows

**DOI:** 10.3389/fvets.2022.1065258

**Published:** 2023-01-05

**Authors:** Damiano Cavallini, Alberto Palmonari, Ludovica Maria Eugenia Mammi, Francesca Ghiaccio, Giorgia Canestrari, Andrea Formigoni

**Affiliations:** Department of Veterinary Medicine, University of Bologna, Bologna, Italy

**Keywords:** undegraded NDF, apparent digestibility, fecal sampling, dry hay-based TMR, dairy cows, rumination time, eating behavior, time-budget

## Abstract

**Introduction:**

The aim of this study was the evaluation of nutrient excretion patterns in samples of feces collected every 2 h to define the best sampling protocol for estimation of apparent digestibility.

**Methods:**

Four multiparous mid-lactation Holstein cows, housed in a tie stall barn and milked twice a day (0800; 1900 h), were enrolled. Dry total mixed ration (TMR) without silages was fed once (0800 h) per day. Feces were sampled every 2 h for 72 h. Each sample was divided in 3 portions: hourly sample sample (8, 10, 12, 14, 16, 18, 20, 22, 00, 2, 4, 6), 8-h composite sample (00–06, 08–14 and 16–22), and a 24-h composite sample. Complete chemical analyses were performed and total tract nutrient digestibility was calculated using undegraded neutral detergent fiber at 240 h of *in vitro* fermentation (uNDF240h) as a marker. Feeding and rumination patterns were also recorded during the trial.

**Results and discussion:**

For some parameters, excretion was not constant throughout the day: neutral detergent fiber (aNDFom), undegraded neutral detergent fiber at 24 h of *in vitro* fermentation (uNDF24h), uDNF240h, total tract crude protein digestibility (TTCPD), total tract neutral detergent fiber digestibility (TTaNDFomD), total tract potentially degraded neutral detergent fiber at 240 h of *in vitro* fermentation digestibility (TTpdNDF240hD) with minimal values after new TMR delivery and maximal values 12 h after feed delivery. Feeding and ruminating behavior seemed to have an important role in the excretion pattern, due to the pushing and evacuating effect they have. Considering our results, two fecal samples at 12 and 24 h after the TMR delivery are suggested. For one daily sample, 12 h post time of most stable and constant rumination 0000–0600 h, which is also 8 h post feed delivery is suggested.

## 1. Introduction

The nutritive value of a ration for bovines is definite by their capability to assume, digest, and metabolize dietary nutrients. To generate correct evaluations of ration digestibility or the nutrients within, classically, the individual 24 h dry matter intake and total fecal collection must be done. The necessity to measure individual dry matter intake is a hands-on challenge for the evaluation of digestibility under commercial farms (1).

Evaluation of fecal composition allows the assessment of the apparent total tract digestibility of fiber, starch, and other nutrients. Indigestible markers such can be used to monitor nutrient digestibility, but knowledge of the concentration of the marker and the nutrient of interest in both the feed and feces is required (2). The indigestible neutral detergent fiber (iNDF) is an intrinsic marker used to estimate the total-tract nutrient digestibility in dairy cows as an alternative to silica or acid insoluble ash (3–8) avoiding total fecal collection (9).

The standard nomenclature throughout the literature refers to iNDF as the residue of the neutral detergent fiber (aNDFom) evaluated *in vivo* after 288 h of ruminal incubation *in situ* or after 240 h of fermentation *in vitro*. To improve the accuracy of the standard terminology used to describe fiber fractions, Mertens (10) coined the term “undigested NDF” (uNDF) which represents the laboratory measure of undigested fiber at a specified fermentation time. The uNDF fraction was estimated *via* long-term (240 h) *in vitro* fermentation (11) or by incubating the samples in bags placed in the rumen for 288 h (6). The acronyms used are respectively uNDF240h and uNDF288h. The uNDF influences the rate of potentially degradable NDF (pdNDF) degradability (12), the gut fill, and the physical effectiveness properties of the forages in inducing chewing and rumination.

The uNDF240h has been used by several researchers to estimate the *in vivo* apparent nutrient total tract digestibility in dairy cows (13–18).

Another analysis of particular interest could be the uNDF estimated at 24 or 30 h of fermentation. This parameter could be used to evaluate the apparent digestibility of the fast pool of the potentially fermentable neutral detergent fiber, another index of rumen functionality (19).

The approaches pronounced for assessing nutrient digestibility mostly depend on using fecal samples that are representative of a daily period, total collected over a number of days, or compositing fecal samples over several time points and obtained from a large number of animals (1). In fact, iIn experimental conditions, multiple time points of fecal collection are used to evaluate the diet digestibility, but sampling and analysis of uNDF240h of many samples are time-consuming and expensive even if NIR analysis is used (20).

In commercial settings in which exploiting nutrient utilization could obviously rise income margins, it is impracticable to continuously collect feces from large numbers of cattle in the herd. Few sampling times from a small number of cattle to produce the same information would be idyllic, but there has been limited research to evaluate the pattern in fecal nutrient excretion over the day (1).

To our knowledge, no information is available to define the optimal sampling frequency of feces for the proper estimation of a diet's apparent digestibility in dairy cattle.

The objective of this paper was to examine how fecal uNDF240h varies with 2 h time point sampling to define the best protocol for estimating apparent total tract nutrient digestibility for research or farm use.

## 2. Materials and methods

This study was conducted at the University of Bologna (Italy) didactic and experimental farm. As every commercial farm, it follows the European Union legal requirements (98/58/EC) (21) concerning the protection of animals kept for farming purposes.

**Figure 1:**
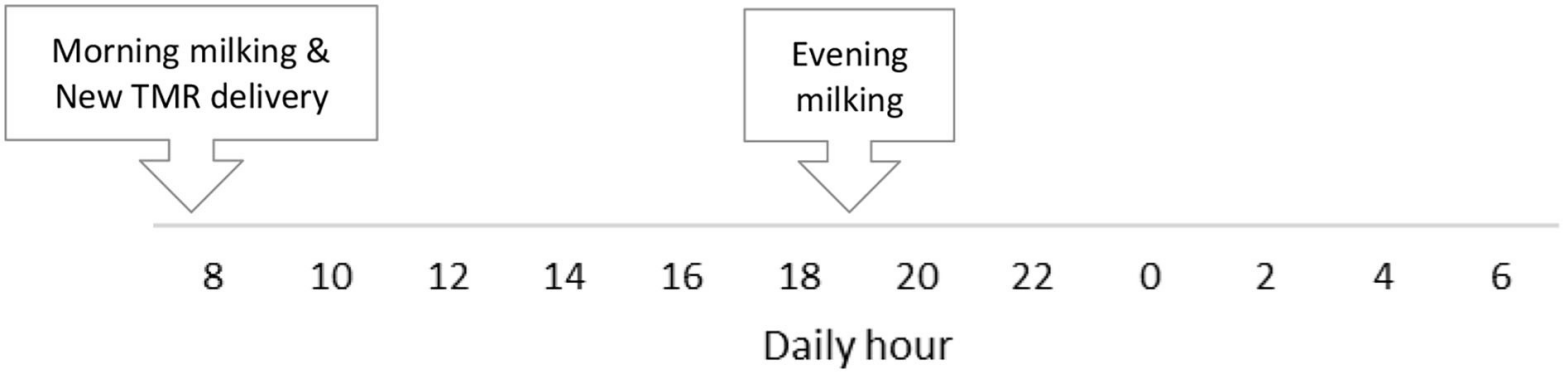
Farm daily routine implemented during the trial.

Four multiparous mid- to late-lactation Holstein cows, homogeneous for body weight (610 ± 50 kg), parity (2.25 ± 0.43), milk production (33.5 ± 4.6 kg/d), and days in milk (210 ± 21) were selected. The study was carried out in December 2016. The farm's daily routine is shown in Figure 1. Animals were kept in a naturally ventilated and lighted tie-stall barn and milked twice a day (0730, and 1,930 h), fed with the same ration delivered as total mixed ration (TMR). Rations were formulated using dry forages and concentrates like the common rations used for Parmigiano Reggiano cheese production. TMR was prepared once a day (Zago Mixer; Padova, IT) and delivered at 0800 h in individual feed bunks able to measure the disappearance of the feed during the day (Dinamica Generale, Poggio Rusco, MN, Italy) thank four digital scales under each one. Cows were fed for *ad libitum* intake (~110% of the expected intake), clear daily and TMR residues were measured every morning before the new distribution. The observational period, lasting 72 h. In particular, feces were collected every 2 h starting at 0800 h on day 1 to 0600 h on d 3, with a total of 36 samples per cow. At each time point, a portion of the sample was pooled, in order to be added to a composite sample every 8 h, and a daily sample. To summarize, the daily time points were: h8, h10, h12, h14, h16, h18, h20, h22, h00, h2, h4, h6; composite samples were: c8-14 (the composite subsamples of h8, h10, h12, h14), c16-22 (the composite subsamples of h16, h18, h20, h22) and c00-06 (the composite subsamples of h00, h2, h4, h6); finally a daily sample 24 h (the composite subsample of all the daily samples). Body weight was recorded daily (Afiweight scale, Afikim, Israel). Dry matter intake (DMI) was determined by recording feed offered and refused for each cow, feeding behavior by the weight variation recorded from the individual manger above described. Drinking water was recorded by an individual water meter. Samples of diets and orts were collected daily and a portion of each sample was dried in a forced-air oven at 105°C for 24 h for DM determination, and at 65°C until stable weight for nutrients determination. Rumination time was measured using the Hi-Tag rumination monitoring system (SCR Engineers Ltd., Netanya, Israel), and analyzed with a resolution of 2 h (22).

### 2.1. Chemical analyses

Daily TMR and fecal samples were analyzed for chemical composition by wet chemistry according to the following methods (23, 24): crude protein (CP) according to AOAC (25) (method 976.06 and 984.13) using a Kjeldahl nitrogen analyzer (Gerhadt Vapodest 50, Gerhardt GmbH, Königswinter, Germany), starch determined according to Ehrman (26) and AOAC (25) (method 920.40), ether extract according to AOAC (25) (method 920.390020), ash-corrected α-amylase–treated neutral detergent fiber (NDF) with the addition of sodium sulfite (aNDFom), acid detergent fiber (ADF) and acid detergent lignin (ADL) according to Mertens et al. (27), ash after 4 h combustion in a muffle furnace at 550°C (Vulcan 3–550, Dentsply Neytech, Burlington, NJ, USA).

The ration particle size was determined using a RoTap Separator (W.S. Tyler, Mentor, OH), and the physically effective (pef) NDF was calculated as the product of aNDFom content and its pef (28). The uNDF at 24 and 240 h of the ration were determined using an *in vitro* fermentation (29) in buffered media containing ruminal fluid according to the procedure described by Palmonari et al. (30).

The uNDF240h was used as a marker to calculate the total tract nutrient digestibility as follows:


$$\begin{array}{l} TTOMD,\;\%\;OM=\;100\;-[\left(dietary\;uNDF240h,\;\%DM/fecal\;uNDF240h,\;\%DM\right)*\\ \;\left(fecal\;OM,\;\%DM/dietary\;OM,\;\%DM\right)]\;\\ \text{TT}CP\text{D},\;\%\;CP\text{ =100 }-[\left(\text{dietary uNDF240h},\;\%\text{DM/fecal uNDF240h},\;\%\text{DM}\right)*\\ \;\left(\text{fecal }CP,\;\%\text{DM/dietary }CP,\;\%\text{DM}\right)]\;\\ \text{TT}Starch\text{D},\;\%\;Starch\text{= 100 }-[\left(\text{dietary uNDF240h},\;\%\text{DM/fecal uNDF240h},\;\%\text{DM}\right)*\\ \;\left(\text{fecal }Starch,\;\%\text{DM/dietary }Starch,\;\%\text{DM}\right)]\\ \text{TT}aNDFom\text{D},\;\%\text{ aNDFom = 100 }-[\left(\text{dietary uNDF240h},\;\%\text{DM/fecal uNDF240h},\;\%\text{DM}\right)*\\ \;\left(\text{fecal }aNDFom,\;\%\text{DM/dietary }aNDFom,\;\%\text{DM}\right)]\\ \text{TT}pdNDF24h\text{D},\;\%\;pdNDF24h\text{ = 100 }-[\left(\text{dietary uNDF240h},\;\%\text{DM/fecal uNDF240h},\;\%\text{DM}\right)*\\ \;\left(\text{fecal }pdNDF24h,\;\%\text{DM/dietary }pdNDF24h,\;\%\text{DM}\right)]\\ \text{TT}pdNDF240h\text{D},\;\%\text{ pdNDF240h = 100 }-[\left(\text{dietary uNDF240h},\;\%\text{DM/fecal uNDF240h},\;\%\text{DM}\right)*\\ \;\left(\text{fecal }pdNDF240h,\;\%\text{DM/dietary }pdNDF240h,\;\%\text{DM}\right)]. \end{array}$$

### 2.2. Statistical analysis

All the data were collected in Excel (31), behavioral data: DMI, eating time, rumination time, and inactive time (calculated as residual time with no eating or ruminating) were summarized in 2 h periods (e.g., h8 means from 0800 to 0959).

Repeated measures data were analyzed with the MIXED procedure for repeated measures of JMP (version 16.1 Pro, Statistical Analysis Systems Institute Inc., Cary, NC) with model effects of time points and cow as experimental unit and random effect. The normal distribution of the data was checked before the analysis (Shapiro–Wilk's test) and after for the resulted residuals. A first-order autoregressive structure was used to model repeated measures on individual animals within each day. Least squares means (LSM) were separated using Tukey's procedure when a significant F-test (*P* ≤ 0.05) was detected. A *P*-value ≤ 0.05 was considered statistically significant, and a *p*-value ≤ 0.01 was considered highly significant. Results were graphically reported in histograms (LSM ± SE).

## 3. Results

Descriptive statistics results (mean ± SD) of the TMR and feces chemical analysis and the average calculation of the apparent total tract digestibility of OM, CP, starch, aNDFom, pdNDF24h, and pdNDF240h are reported in Table 1.

**Table 1:** Composition of TMR diet and feces and apparent total tract digestibility (mean ± SD).

	**TMR^a^**	**Feces**
DM, %	87.15 ± 1.03	12.95 ± 1.10
Ash, %DM	6.82 ± 0.52	10.03 ± 1.41
CP, %DM	14.53 ± 1.08	13.65 ± 1.41
Starch, %DM	22.39 ± 2.02	2.85 ± 0.95
aNDFom^b^, %DM	37.86 ± 2.86	62.30 ± 2.79
ADF, %DM	24.62 ± 1.36	44.97 ± 2.12
ADL, %DM	5.50 ± 0.75	20.89 ± 4.96
uNDF${}_{24\text{h}}^{\text{c}}$, %DM	19.78 ± 2.24	54.03 ± 2.74
uNDF${}_{240\text{h}}^{\text{d}}$, %DM	11.63 ± 1.36	34.86 ± 3.13
peNDF^e^, %DM	19.52 ± 2.18	-
peuNDF^f^, %DM	6.01 ± 0.79	-
TTOM^g^D, %OM^h^	-	67.79 ± 3.09
TTCPD, %CP^i^	-	68.66 ± 4.03
TTStarchD, %Starch^j^	-	95.75 ± 1.24
TTaNDFomD, %aNDFom^k^	-	45.10 ± 2.12
TTpdNDF_24h_D, %pdNDF${}_{24\text{h}}^{\text{l}}$	-	84.74 ± 4.18
TTpdNDF_240h_D, %pdNDF${}_{240\text{h}}^{\text{m}}$	-	65.10 ± 5.19

^*a*^TMR Ingredients: 38.0% grass hay [the quality of the hay was checked according to Cavallini et al. (32)], 4.0% straw, 24.0% steam-flaked corn, 4.0% cane-beet molasses blend^*n*^, and 30.0% feed mix [29%af sorghum meal, 29%af wheat bran, 22%af soybean meal (44%CP), 15%af soy full fat flaked, 2%af calcium carbonate, 1%af sodium bentonite, 1%af NaCl, 0.25%af magnesium oxide, 0.24%af microminerals, 0.01%af vitamins ADE.]. ^*b*^Amylase- and sodium sulfite-treated NDF with ash correction. ^*c*^Undigestible NDF estimated via 24-h *in vitro* fermentation. ^*d*^Undigestible NDF estimated via 240-h *in vitro* fermentation. ^*e*^Physically effective NDF (aNDFom^*^pef), calculated using the Ro-Tap system (28). ^*f*^Physically effective uNDF_240*h*_ (uNDF${}_{240h}^{*}$pef), calculated using the Ro-Tap system (33). ^*g*^Organic matter (100 – ash). ^*h*^Apparent OM total tract digestibility. ^*i*^Apparent CP total tract digestibility. ^*j*^Apparent starch total tract digestibility. ^*k*^Apparent aNDFom total tract digestibility. ^*l*^Apparent potentially digestible NDF_24*h*_ (pd NDF_24*h*_)total tract digestibility. ^*m*^Apparent potentially digestible NDF_240*h*_ (pd NDF_240*h*_)total tract digestibility. ^*n*^Characterized according to Palmonari et al. (34).

**Figure 2:**
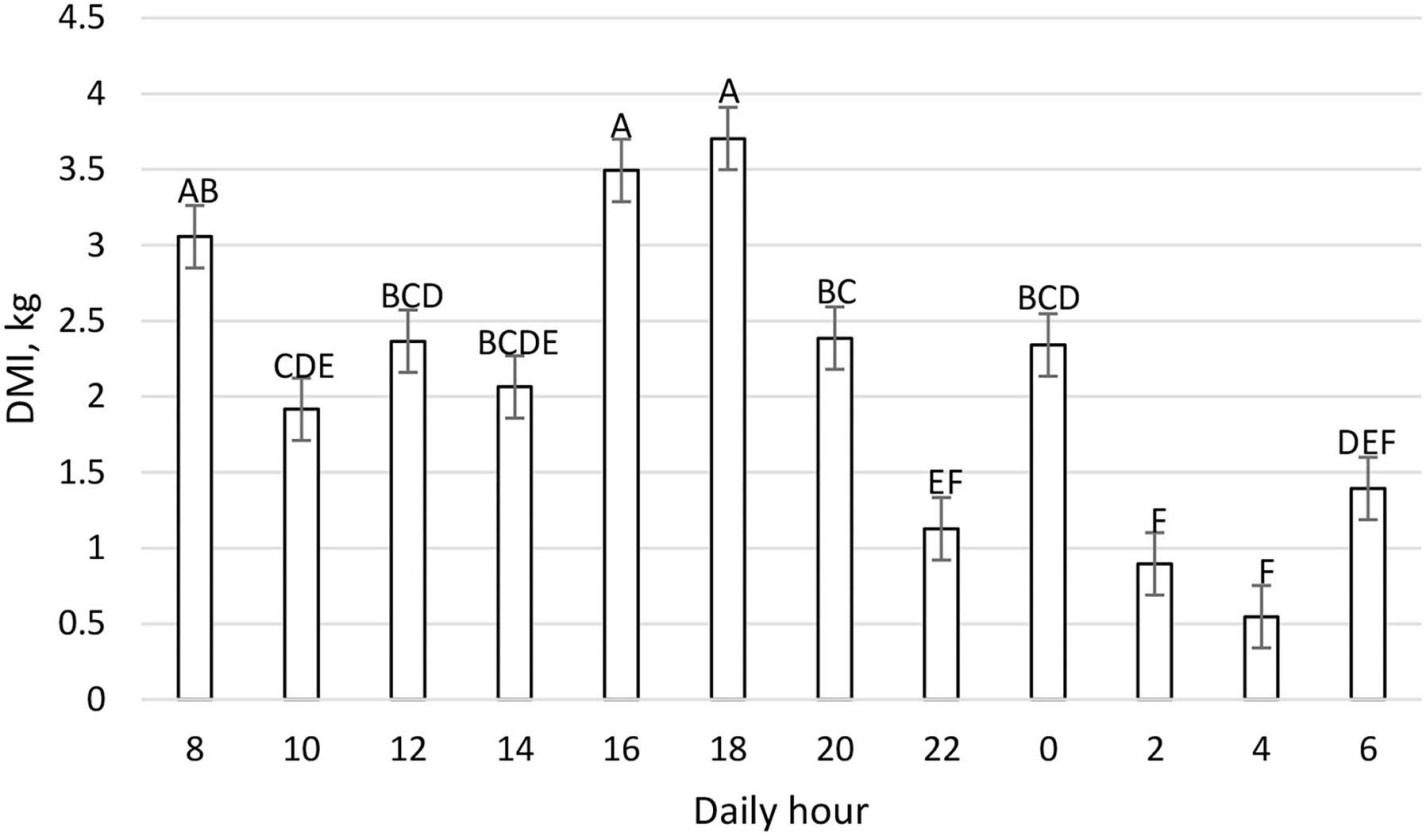
Daily DMI (kg) pattern (LSM ± SE) on a 2 h basis during the fecal sampling period. Letters for differences ≤ 0.05. Each time point refers to 2 h intake. *P-value* < 0.01; SEM = 0.21.

**Figure 3:**
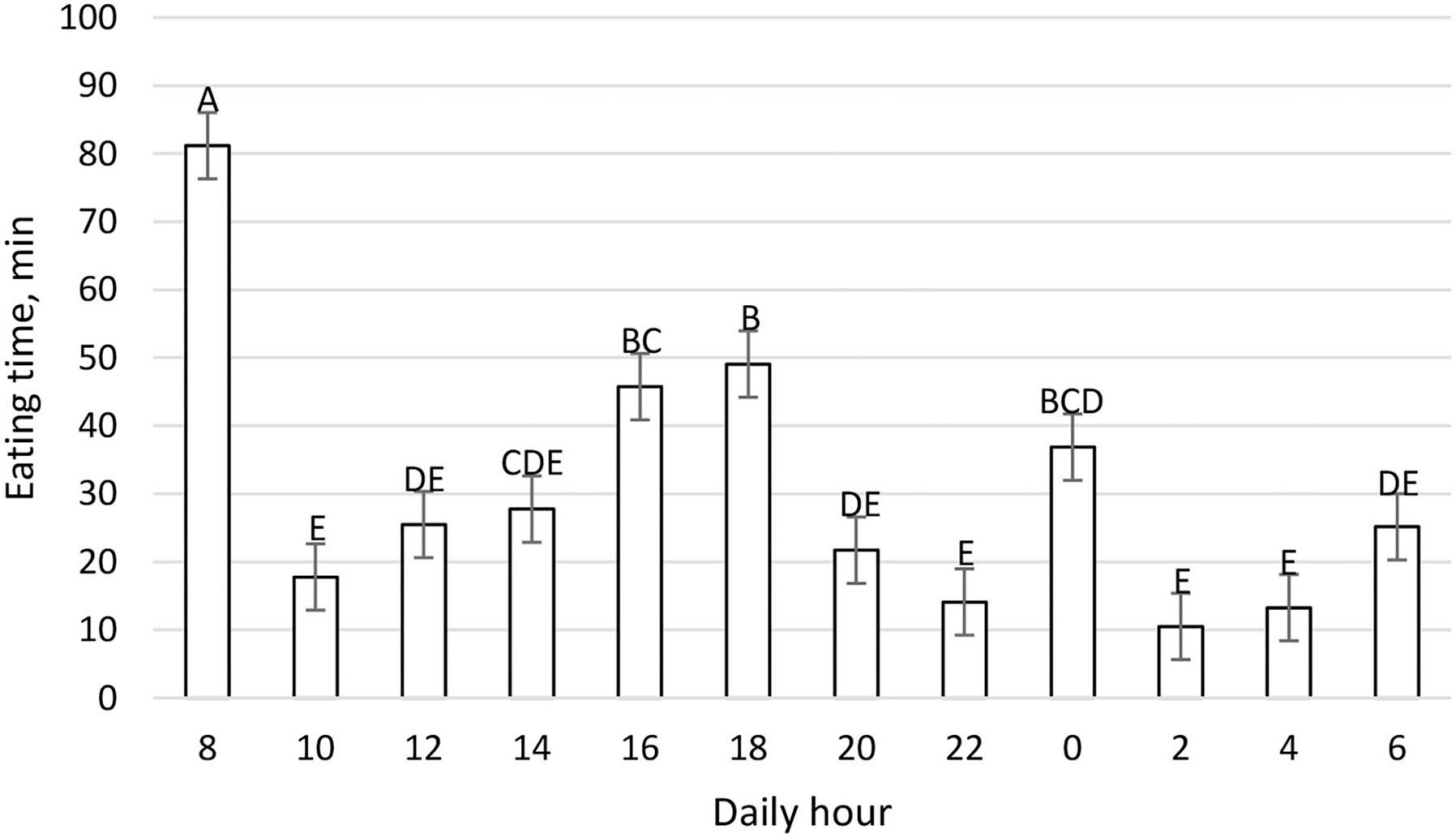
Daily eating time (min) pattern (LSM ± SE) on a 2 h basis during the fecal sampling period. Letters for differences ≤ 0.05. Each time point refers to 2 h eating time. *P-value* < 0.01; SEM = 4.87.

**Figure 4:**
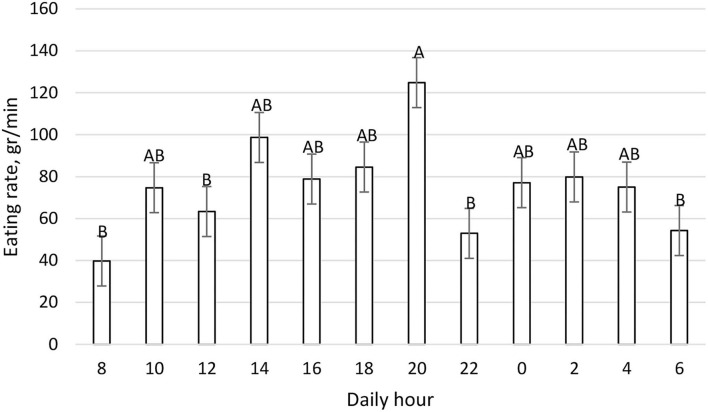
Daily DMI rate (grams/min) patter (LSM ± SE) on a 2 h basis during the fecal sampling period. Letters for differences ≤ 0.05. Each time point refers to 2 h DMI rate. *P-value* < 0.01; SEM = 11.92.

**Figure 5:**
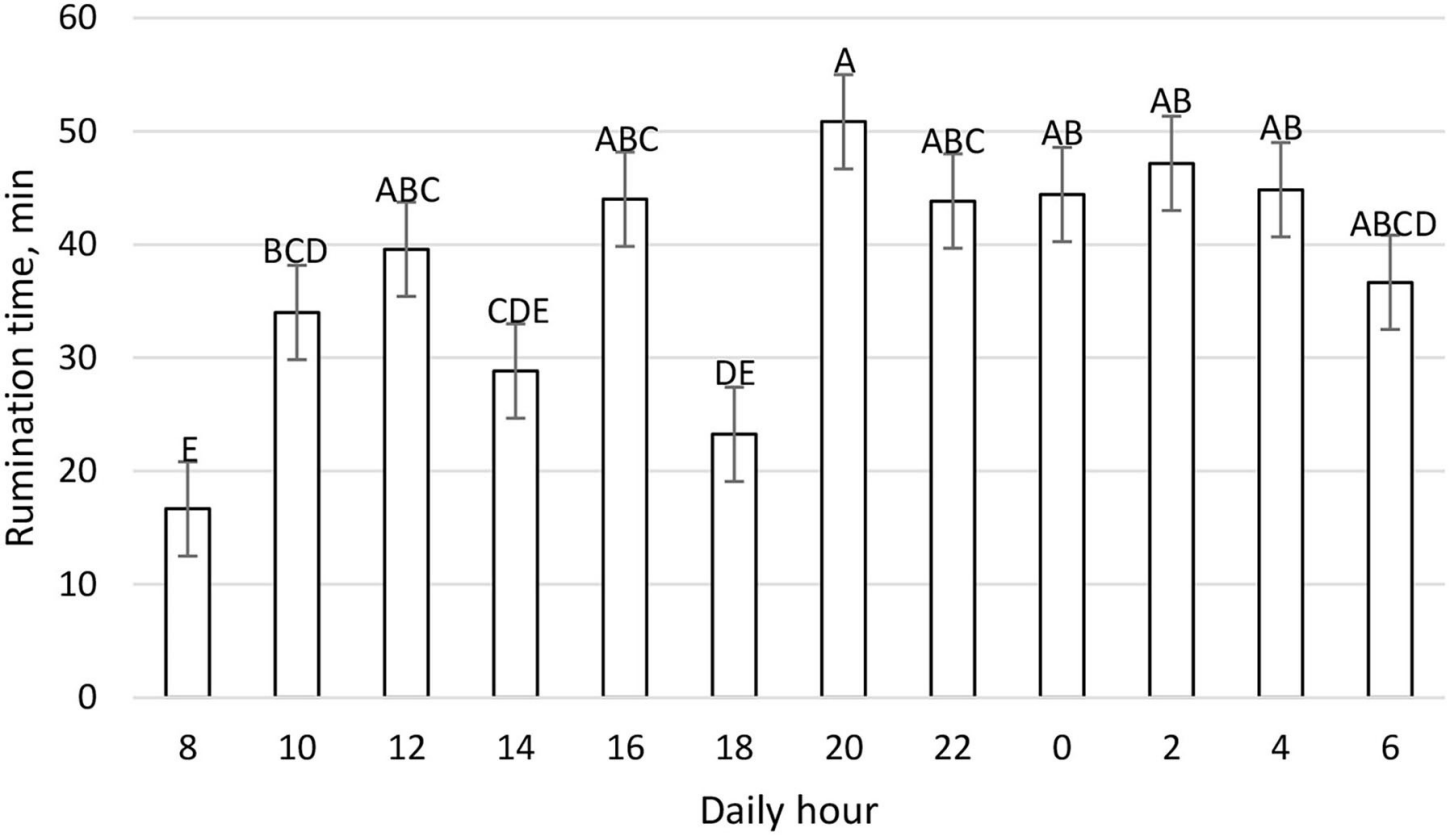
Daily rumination time (min) patter (LSM ± SE) on a 2 h basis during the fecal sampling period. Letters for differences ≤ 0.05. Each time point refers to 2 h rumination. *P-value* < 0.01; SEM = 4.16.

**Figure 6:**
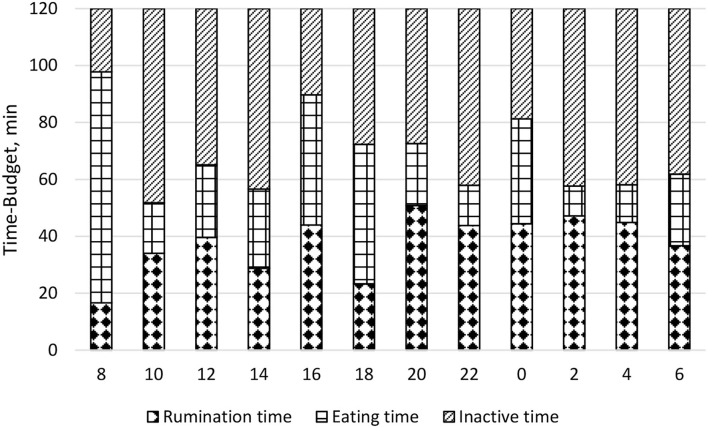
Daily cows' time-budget (min) on 2 h bases during the fecal sampling period. Rumination time: time spent ruminating; Eating time: time spent eating; Inactive time: time spent without ruminating or eating. Time-budget display was made according to Raspa et al. (35).

The mean daily DMI was 25.29 ± 1.00 kg and the daily pattern is shown in Figure 2. New TMR was delivered at 0800; however, cows' peak intake was recorded primarily between 1,600 and 1,959, and secondarily between 0800 and 0959. On the contrary, cows spent more time eating after the new TMR was delivered instead of during the period of higher DMI (Figure 3). The maximum eating rate was recorded between 2,000 and 2,159 h and the minimum between 0800 and 0959 h (Figure 4). The mean daily rumination time was 465.85 ± 85.65 min and followed an opposite pattern to the DMI, highlighting the night period as preferred to ruminate by the enrolled cows (Figure 5). Figure 6 shows the time budget during the sampling period: the “inactive time” was greater late in the morning (1,000–1,459 h) and during the night (2,200–0759 h).

The mean daily water intake was 134.61 ± 25.67 l.

**Figure 7:**
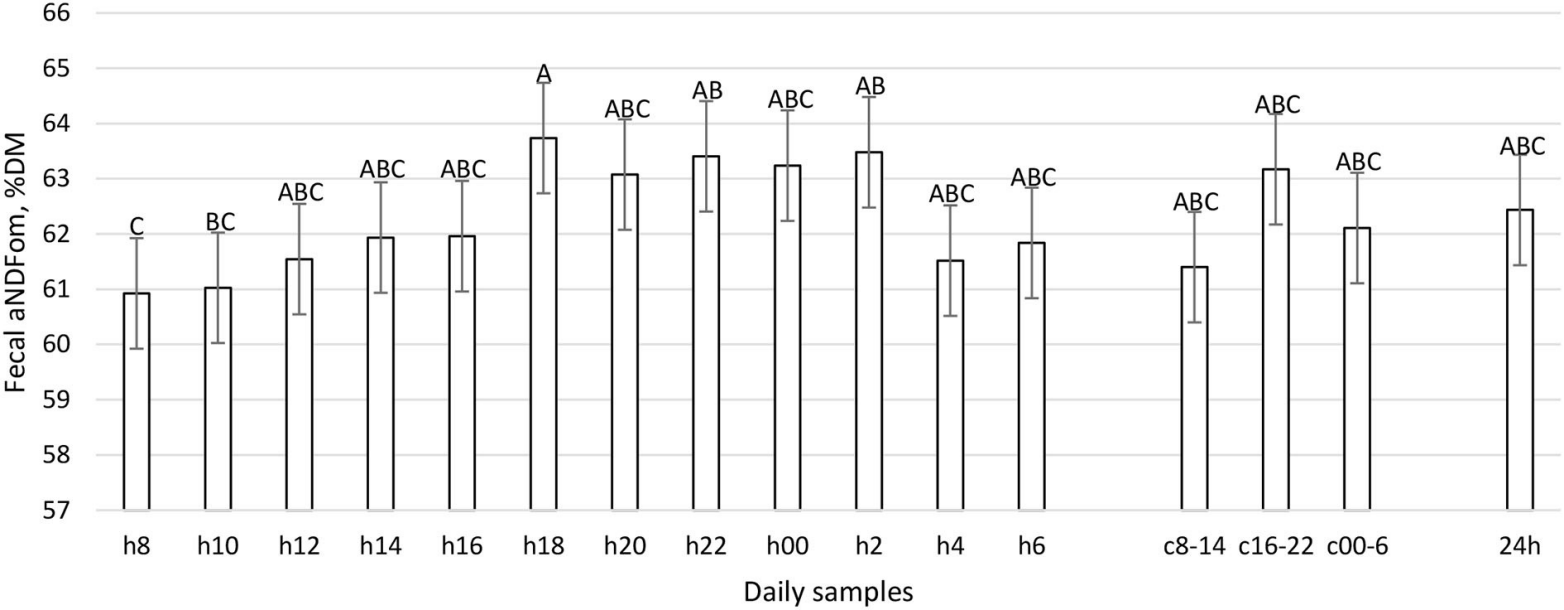
Fecal aNDFom (%DM) excretion (LSM ± SE) in sampled time points (daily samplings: h8, h10, h12, h14, h16, h18, h20, h22, h00, h2, h4, h6; composites: c8-14, c16-22, c00-6, 24h). Letters for differences ≤ 0.05. *P-value* < 0.01; SEM = 0.80.

**Figure 8:**
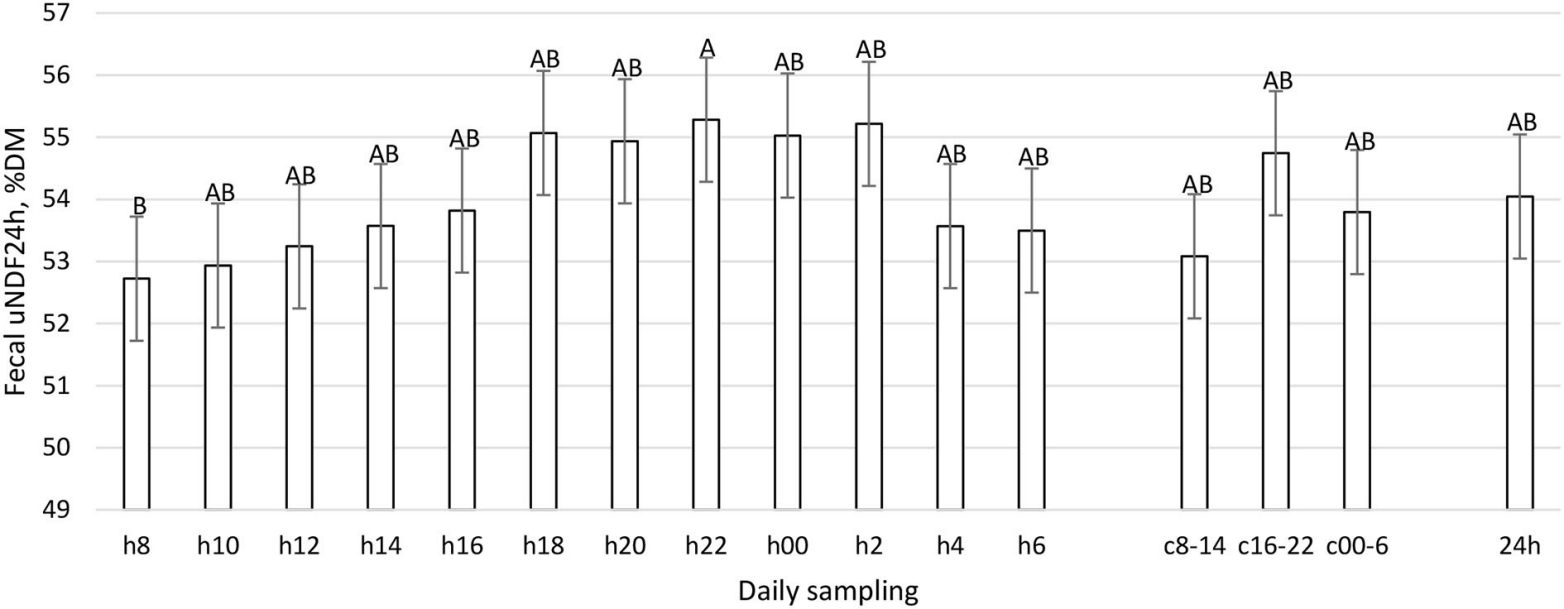
Fecal uNDF24h (%DM) excretion (LSM ± SE) in sampled time points (daily samplings: h8, h10, h12, h14, h16, h18, h20, h22, h00, h2, h4, h6; composites: c8-14, c16-22, c00-6, 24h). Letters for differences ≤ 0.05. *P-value* < 0.01; SEM = 0.76.

**Figure 9:**
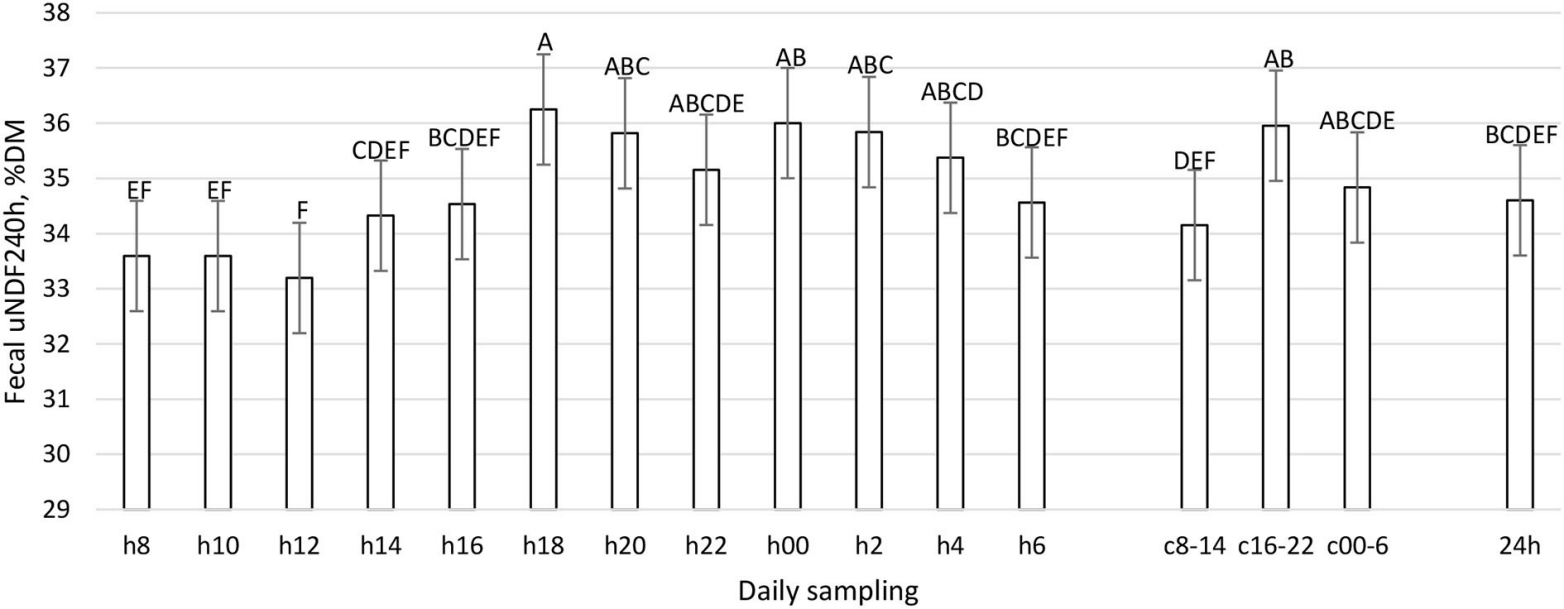
Fecal uNDF240h (%DM) excretion (LSM ± SE) in sampled time points (daily samplings: h8, h10, h12, h14, h16, h18, h20, h22, h00, h2, h4, h6; composites: c8-14, c16-22, c00-6, 24 h). Letters for differences ≤ 0.05. *P-value* < 0.01; SEM = 0.88.

The daily excretion of fecal ash, CP, and starch was constant during the day (*P* > 0.05, Table 1), while aNDFom, uNDF24h, and uDNF240h varied according to the daily hour (*P* < 0.01, Figures 7–9). The maximum fecal excretion of aNDFom was recorded at 1,800, while minimum values were at 0800 and 1,000. Other time points reported mean values comparable to composites samples. A similar pattern was observed in the fecal uNDF24h, with a minimum at 0800 and other time points equivalent to the composite samples. In contrast, fecal uNDF240h excretion was more variable throughout the day. Minimum excretion was recorded between 0800 and 1,200 while maximum excretion was during the evening and the nighttime (1,800, 2,000, 0000, 0200, 0400). Composite samples reflected the mean values of the time points that comprised the composites. The daily composite sample (24 h) was similar to the h14, h16, and h6 samples.

**Figure 10:**
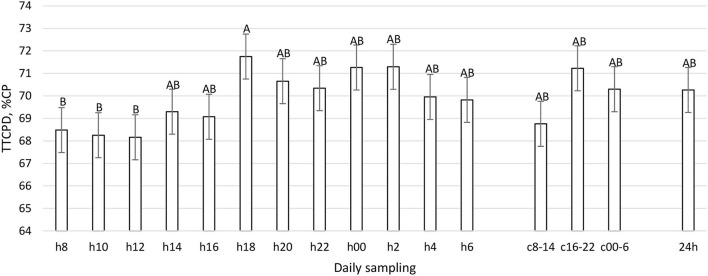
Total Tract CP Digestibility (%CP) (LSM ± SE) in sampled time points (daily samplings: h8, h10, h12, h14, h16, h18, h20, h22, h00, h2, h4, h6; composites: c8-14, c16-22, c00-6, 24 h). Letters for differences ≤ 0.05. *P-value* < 0.01; SEM = 1.50.

**Figure 11:**
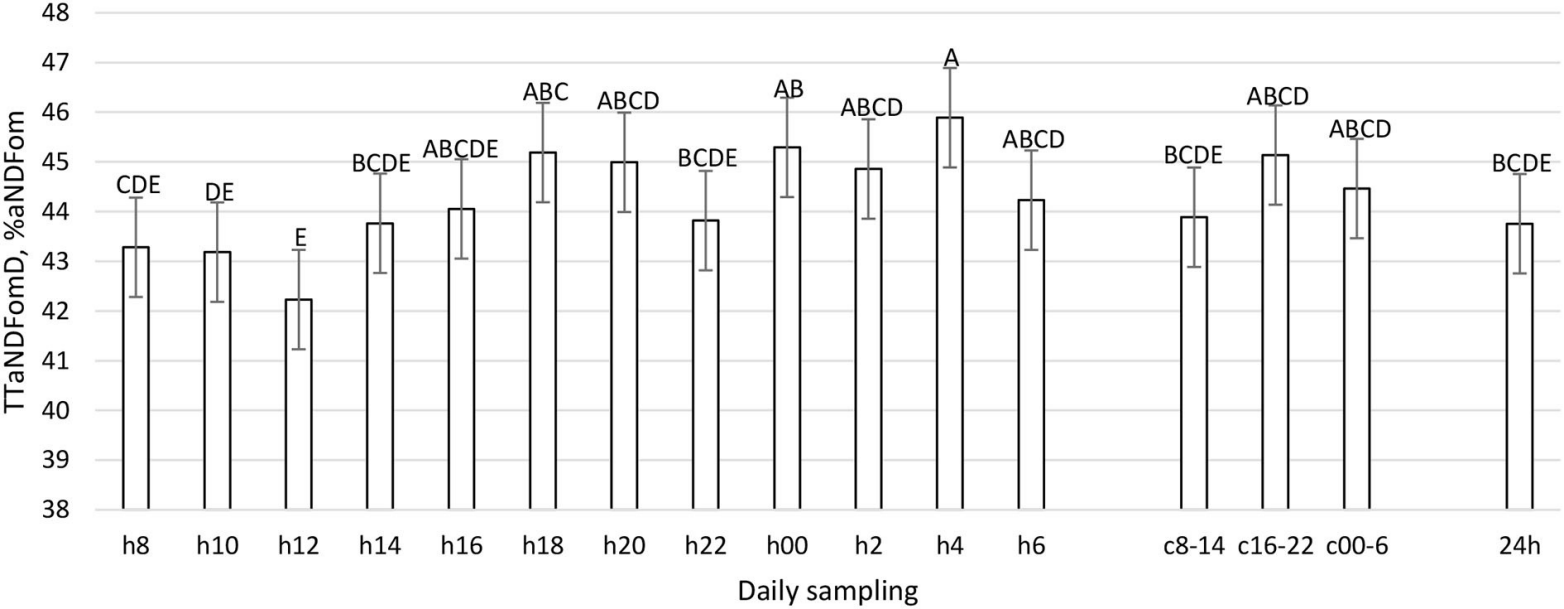
Total Tract aNDFom Digestibility (%aNDFom) (LSM ± SE) in sampled time points (daily samplings: h8, h10, h12, h14, h16, h18, h20, h22, h00, h2, h4, h6; composites: c8-14, c16-22, c00-6, 24 h). Letters for differences ≤ 0.05. *P-value* = 0.03; SEM = 0.74.

**Figure 12:**
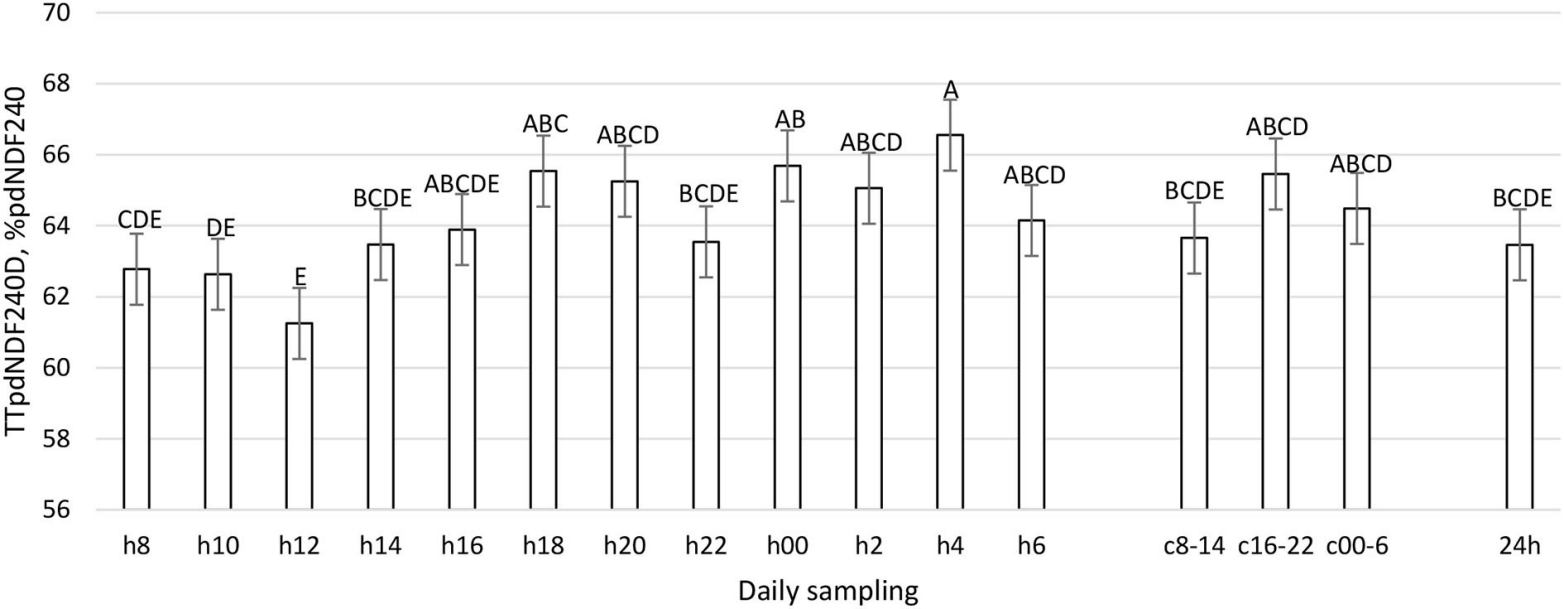
Total Tract pdNDF_240h_ Digestibility (%pdNDF_240h_) (LSM ± SE) in sampled time points (daily samplings: h8, h10, h12, h14, h16, h18, h20, h22, h00, h2, h4, h6; composites: c8-14, c16-22, c00-6, 24 h). Letters for differences ≤ 0.05. *P-value* = 0.04; SEM = 1.13.

The daily pattern of apparent total tract nutrient digestibility was constant for OM, starch, and pdNDF24h (*P* > 0.05, Table 1), while the CP, aNDFom, and pdNDF240h patterns were significantly influenced by sampling time (*P* < 0.01, Figures 10–12). In TTCPD, TTaNDFom, and TTpdNDF240hD a lower digestibility was recorded between 0800 and 1,200, while the highest values were recorded in different time points between h16 and h4, according to the different parameters. Composite samples (c8-14, c16-22, and c00-6) properly reflected the daily pattern. Finally, the daily composite (24 h) was comparable to the samples taken at 1,400, 1,600, 2,200, and 0600.

## 4. Discussion

This work dealt with the investigation of fecal nutrient excretion patterns and feeding behavior in mid to late-lactation dairy cows fed dry hay-based TMR and kept in a tie-stall barn.

The TMR fed was adequate in terms of nutrient composition (36), and particle size (peNDF = 19.52 ± 2.18% of DM) was in line with previous studies based on similar rations (37–40). Fecal composition was normal for a cow in this lactation phase and the apparent total tract digestibility of the different nutrients indicates a good level of digestion (Table 1). These apparent total tract digestibility results are comparable to other published work with lactating dairy cattle (17, 41, 42).

Figure 1 shows the feeding behavior recorded during the trial, underlining how the cow eats throughout the whole day, and confirming *ad-libitum* intake. Despite our expectations, the delivery of new TMR (0800 h) did not elicit a higher intake, and in fact, cows preferred to eat more during the afternoon. Previous work (43) reported that new TMR delivered produced the largest meal of the day. This difference could be related to the hour of the delivery: in this trial, TMR was delivered at 0800 h while in Heinrichs et al. (43), it was delivered at 1,900 h. Moreover, the DIM could influence the meal eating behavior. For this study, mid-lactation cows instead of early lactation cows were used. Milk yield could also impact this daily pattern, but other conditions such as dietary forages, milking routine, and environmental conditions were the same in this study. Interestingly, the time spent eating was not directly correlated to the intake. In fact, the new TMR raised more interest from the cows, which spent more time eating, while during the afternoon cows ate a greater amount at a faster rate (Figure 4). This aspect highlights how the farm's daily routine could deeply influence cows' behavior.

Recorded rumination time had physiological values according to common references (44, 45). The daily rumination time pattern, shown in Figure 5, indicates that the cow ruminates constantly during the day, except at 0800 and 1,800 h during which the cow spent more time eating (Figures 2, 3). Results of the daily rumination time pattern are comparable to what was reported by a previous study (43).

Interestingly, if the time spent ruminating was added to the time spent eating, we did not obtain a constant pattern of chewing during the day (Figure 6). We can hypothesize that this fact is probably related to the time spent sleeping or doing nothing (46).

To the best of our knowledge, this work represents the first time in which the pattern of fecal nutrient excretion was studied with so frequent sampling in dairy cattle. The rationale was to study the curve of nutrient excretion and identify the best time points to perform focused sampling protocols, particularly at the farm level. The fecal residual nutrient excretion is not constant during the day for many parameters (Figures 7–12). To summarize the findings, for the considered parameters, the common minimum was often recorded between 0800 and 1,200 h, corresponding to a new TMR delivery, and the maximum between 1,800 and 0400 h, starting from 10 to 12 h after new TMR delivery, with some differences between studied parameters. These differences could be related to feeding or ruminating behavior (19, 42, 47).

The sinusoidal excretion pattern observed over the day is possible a reflection of the eating bouts consumed over the time. Passage rate of digesta through the foreign stomachs is triggered by particle size, rumen washout, rumen wall distension or papillae tactile signals that occur subsequently each intake (48). When there is minimal competition for feed, pastured, feedlot, group (49), and individually fed (50) cattle typically exhibit a biphasic feeding pattern over the day. The first meal occurs just after the time of first feed delivery, with subsequent secondary meals (46, 51).

If we compare Figures 1–6 with Figures 7–12, the minimum of fecal nutrient excretion occurs right after the new TMR delivery (0800 h) whereas the maximum excretion starts after the large afternoon meal (1,600–1,959 h), corresponding to the maximum rumination time period (2,000–0559 h). We can speculate that one reason that could explain our results is the “pushing effect” of the new feed intake that expands the rumen and promotes fecal evacuation (47). A second explanation is related to the rumen contraction cycles that favor rumen evacuation and passage of less fermented or digested feed (52, 53). In fact, fodder evacuation is more related to contractions of the reticulum and rumen, along with regurgitation and mastication, and consequently, the longer retention time results in a more uniform passage rate and fecal excretion pattern (1). This assumption is in accordance with Miller et al. (42), who reported that greater DMI was related to a faster NDF turnover rate. At the same time, the minimum uNDF240h excretion occurs in the morning, just after the night period, when the enrolled cows consumed a minimal amount of TMR.

Interestingly the excretion of uNDF24h has the same pattern as uNDF240h indicating that the evacuation of the fast pool fiber fraction is not different.

Considering the results of this experiment, our suggestion to study the minimum and maximum excretion of the different nutrients is to sample in both these time periods (0800–1,200 and 1,800–2,000 h). In order to identify the best time-point to have a representative value in terms of nutrient excretion and digestibility, we compared the different sampling time points with 8 h composites (c8-14, c16-22, and c00-6) and daily composites (24 h).

The results obtained show that 8-h composite samples reflect the average values of single time points. This finding is very important in the future perspective to reduce the number of analyses and relative cost while obtaining representative digestibility results. The composite of all the samples (24 h) represents the whole day and did not differ with the time points of h14, h16, and h6; therefore, these time points could be considered representative of the average daily situation in terms of fecal nutrient excretion and digestibility. These time points (h14 and h16) correspond to 12 h post time of most stable and constant rumination (0000–0600 h), which is also 8 h post new TMR delivery is suggested. Moreover, the main differences between 24 h and the singular time points were found with h8, h10, and h12, but also with h18 and h20.

In the dairy cattle industry, cows are commonly fed up to twice a day, with the first meal delivered shortly after sunrise. Competition for the TMR can be intense, with the first meal of some cows being delayed if the bunk space is limiting (54). Nevertheless, assuming that feed is not restricted, fecal excretion patterns of these cows should be similar of obtained in this research.

From a practical perspective, it would be desirable to collect fecal pats from dairy cattle early in the early afternoon 8 h after the first feeding, as the more daily representative fecal output was observed.

The limitations of this study were the use of the low number of cows in mid- to late-lactation (200 DIM), cows kept in a tie-stall barn, and fed once a day. Further studies must be planned under a wider range of feeding and management conditions in order to evaluate the consistency of the obtained digestibility results.

## 5. Conclusions

The data obtained in this trial showed that the pattern of fecal excretion is not constant throughout the day. Feed intake and rumination behavior seem to be relevant to explain these variations. Considering our results, two fecal samples at 12 and 24 h after the TMR delivery are suggested. For one daily sample, 12 h post time of most stable and constant rumination 0000–0600 h, which is also 8 h post feed delivery is suggested.

However, these data are based on once daily TMR delivery, in mid- to late-lactation cows in a non-competitive feeding status and results must be confirmed in future trials under a wider range of conditions.

## Data availability statement

The raw data supporting the conclusions of this article will be made available by the authors, without undue reservation.

## Ethics statement

Ethical review and approval was not required for the animal study because this study was conducted at the University of Bologna (Italy) didactic and experimental farm. As every commercial farm, it follows the European Union legal requirements (98/58/EC) concerning the protection of animals kept for farming purposes.

## Author contributions

FG and GC performed the laboratory work. DC performed data analysis. DC and AF wrote the manuscript. LM and AP assisted with editing the manuscript. All authors have read and agreed to the final version of the manuscript.
